# Inhibition of Y1 receptor signaling improves islet transplant outcome

**DOI:** 10.1038/s41467-017-00624-2

**Published:** 2017-09-08

**Authors:** Kim Loh, Yan-Chuan Shi, Stacey Walters, Mohammed Bensellam, Kailun Lee, Katsuya Dezaki, Masanori Nakata, Chi Kin Ip, Jeng Yie Chan, Esteban N. Gurzov, Helen E. Thomas, Michaela Waibel, James Cantley, Thomas W. Kay, Toshihiko Yada, D. Ross Laybutt, Shane T. Grey, Herbert Herzog

**Affiliations:** 1 Neuroscience Division, Garvan Institute of Medical Research, St Vincent’s Hospital, Sydney, NSW 2010 Australia; 2 St. Vincent’s Institute of Medical Research, Fitzroy, VIC 3065 Australia; 3 Faculty of Medicine, UNSW Australia, Sydney, NSW 2052 Australia; 4 Immunology Division, Garvan Institute of Medical Research, St Vincent’s Hospital, Sydney, NSW 2010 Australia; 5 Diabetes and Metabolism Division, Garvan Institute of Medical Research, St Vincent’s Hospital, Sydney, NSW 2010 Australia; 6 Department of Physiology, Jichi Medical University, Tochigi, 329-0498 Japan; 7 Present Address: Institute of Experimental and Clinical Research, Pole of Endocrinology, Diabetes and Nutrition, Université Catholique De Louvain, Brussels, B-1200 Belgium; 8 Present Address: Department of Physiology, Anatomy and Genetics, University of Oxford, Oxford, OX1 3PT UK

## Abstract

Failure to secrete sufficient quantities of insulin is a pathological feature of type-1 and type-2 diabetes, and also reduces the success of islet cell transplantation. Here we demonstrate that Y1 receptor signaling inhibits insulin release in β-cells, and show that this can be pharmacologically exploited to boost insulin secretion. Transplanting islets with Y1 receptor deficiency accelerates the normalization of hyperglycemia in chemically induced diabetic recipient mice, which can also be achieved by short-term pharmacological blockade of Y1 receptors in transplanted mouse and human islets. Furthermore, treatment of non-obese diabetic mice with a Y1 receptor antagonist delays the onset of diabetes. Mechanistically, Y1 receptor signaling inhibits the production of cAMP in islets, which via CREB mediated pathways results in the down-regulation of several key enzymes in glycolysis and ATP production. Thus, manipulating Y1 receptor signaling in β-cells offers a unique therapeutic opportunity for correcting insulin deficiency as it occurs in the pathological state of type-1 diabetes as well as during islet transplantation.

## Abstract (web summary)

Islet transplantation is considered one of the potential treatments for T1DM but limited islet survival and their impaired function pose limitations to this approach. Here Loh *et al*. show that the Y1 receptor is expressed in β- cells and inhibition of its signalling, both genetic and pharmacological, improves mouse and human islet function.

## Introduction

Insulin is the key hormone that regulates glucose metabolism and its release from β-cells is tightly controlled by homeostatic mechanisms^1^. Dysregulated release of insulin in response to changes in physiological glucose is central to the pathophysiology of type-1 (T1D) and type-2 (T2D) diabetes^2–4^, thus efforts to increase insulin secretion in response to physiological demands represents a significant area of interest. Currently, islet transplantation is being explored as a potential treatment option for T1D patients to become insulin independent^5^. However, the efficiency of this approach remains poor due to the low survival rate and diminished efficiency of donor islets^6^ further aggravated by the scarcity of donor organs. Therefore, it is recognized that any improvement in this therapeutic option will require a methodology that enhances β-cell function and survival during transplantation. Efforts to achieve this have been mainly directed at the pathways that stimulate insulin secretion, however, with limited success. Although much is known about what inhibits insulin secretion, improving β-cell function by targeting the pathways that suppress the release of insulin remain largely unexplored as a therapeutic option.

Interestingly, it has been demonstrated that upregulation of intracellular cyclic adenosine monophosphate (cAMP) can promote insulin release and improve pancreatic β-cell function^7–11^. For instance, increasing cAMP levels, as a result of glucagon-like-peptide 1 (GLP-1) signaling in β-cells potentiates insulin secretion^12^, highlighting this pathway as a critical control point. Importantly, Y-receptors (Y1, Y2, Y4, Y5, and y6)^13, 14^ which are activated by neuropeptide Y (NPY) family members preferentially associate with G_i/o_ G-proteins and, therefore, act in an inhibitory fashion reducing cAMP levels^15–17^. Particularly, Y1 receptors are highly expressed in β-cells, indicating the potential for an inhibitory effect of Y1 receptor signaling to directly regulate insulin release from β-cells.

Moreover, the other Y-receptor ligands, peptide YY (PYY) and pancreatic polypeptide, are found to be expressed in L-type cells of the gut as well as various pancreatic cell types^18, 19^. PYY released in the gut in response to food ingestion is well recognized for its role to inhibit feeding and increase energy expenditure through the activation of hypothalamic and brain stem Y-receptors^20–23^. Importantly, PYY has also been implicated in the inhibition of insulin release and this notion was supported by studies showing that application of PYY decreases glucose-stimulated insulin secretion from rat and mouse islets^21, 24, 25^. Although these results suggest that PYY may act through a paracrine mechanism to tonically inhibit insulin secretion in the islets, the precise Y receptor mediating this is unclear. Here, we demonstrate that PYY suppressed insulin secretion via Y1 receptor and inhibition of Y1 receptor signaling in islets is advantageous for the enhancement of β-cell function. Our findings have direct relevance to the clinical scenario of islet transplantation and could potentially serve as a new therapeutic option to enhance the efficiency and efficacy of islets derived from deceased organ donors, but also alternate sources including xenogeneic or stem-cell-derived islets.

## Results

### Y1 receptor signaling in β-cells controls insulin secretion

**Fig. 1:**
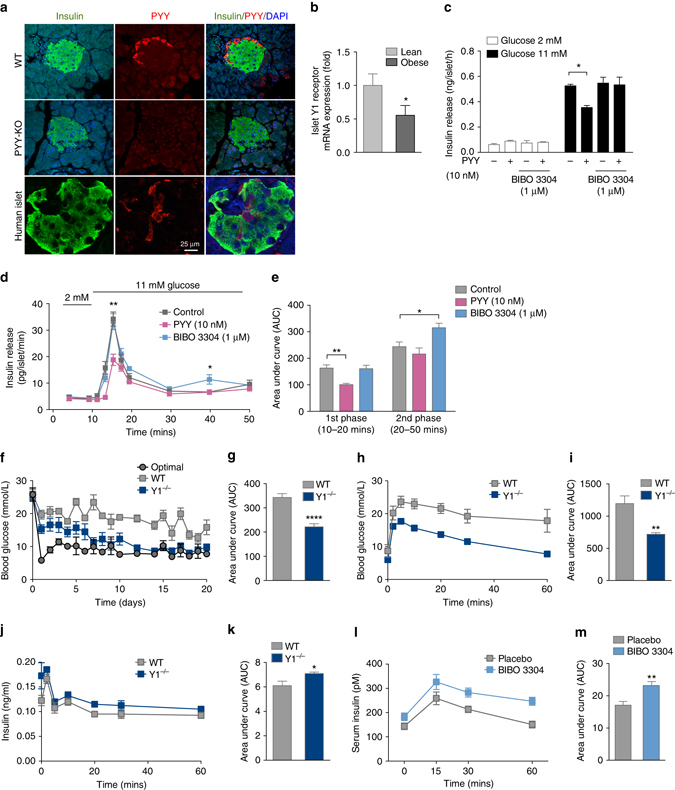
Improved glycemic control in diabetic mice transplanted with Y1 receptor-deficient islets. **a** Photomicrograph showing immune fluorescence staining for insulin (*green*), PYY (*red*) and DAPI (*blue*) in islets from human, wild type, and PYY knockout mice. *Scale bar*, 25 μm. **b** Y1 receptor mRNA expression in pancreatic islets isolated from lean or obese mice determined by qRT-PCR (*n* = 4). **c** Pancreatic islets from C57BL/6JAusb mice were isolated and cultured. Insulin secretion was determined in response to 2 and 11 mM glucose in the presence of PYY and/or Y1 receptor antagonist BIBO3304 (*n* = 3). **d** Perifusion experiment to record insulin release over a 50 min period using islets from C57BL/6JAusb mice stimulated with 11 mM glucose and treated with PYY or Y1 antagonist BIBO3304. (*n* = 4–5). **e** Results expressed as area under the curve. **f** Alloxan-induced diabetic mice were transplanted with an optimal number (300) of WT islets (*n* = 8) or a minimal number (60) from WT islets (*n* = 15) or Y1^−/−^ islets (*n* = 13) and blood glucose levels were monitored for 20 days. **g** Results expressed as area under the curve. **h**–**k** Diabetic mice receiving 60 WT (*n* = 4) or Y1^−/−^ (*n* = 4) islets were fasted overnight and i.v. glucose tolerance tests (1 g/kg body weight) were performed at day 5 post-transplant. Blood glucose levels and insulin production during glucose tolerance tests were monitored. Results are expressed over the time course and as area under the curve. **l**, **m** 7-week-old C57BL/6JAusb mice were treated with placebo (*n* = 6) or with 0.5 μM of the Y1 receptor specific antagonist BIBO3304 (*n* = 6), 2 h prior to i.p. glucose injection (1 g/kg body weight) and serum insulin levels in response to glucose administration at indicated time points were determined. Results are also expressed as area under the curve. Data are shown as mean ± s.e.m. *P < 0.05, **P < 0.01, calculated by *t*-test (**b**, **c**) or two-way ANOVA analysis

In addition to L-type cells, PYY is also expressed in α-cells of mouse and human pancreatic islets^26, 27^ (Fig. 1a), allowing for a possible paracrine action of PYY signaling via Y1 receptors expressed on β-cells to inhibit cAMP production, thereby reducing insulin release. Previous research has shown that genetic deletion of PYY or Y1 receptor genes in mice leads to a phenotype with increased serum insulin levels^21, 28^. Consistent with these findings the overproduction of PYY in transgenic mice results in the opposite phenotype, with reduced serum insulin levels^29^. Using a β-cell specific ‘translating ribosome affinity purification’ mouse model we could show specific enrichment of Y1 receptor mRNA in β-cells confirming the physical localization of this Y-receptor specifically in β-cells (Supplementary Fig. 1a). To verify the purity of this preparation we also performed quantitative reverse transcription PCR (qRT-PCR) on other β-cell specific genes including *Ins1, Mafa1, Pdx1, Nkx6.1* and as a negative control *Pyy* all showing the expected enrichment or absence, respectively (Supplementary Fig. 1b). To further investigate whether Y1 receptors in β-cells are under a regulatory control depending on energy status, we determined Y1 receptor expression in the β-cells from lean and 12-week high-fat diet fed obese mice. Interestingly, we found that Y1 receptor expression was significantly downregulated in obese hyperinsulinemic mice (Fig. 1b) indicating that reduced Y1 receptor signaling may be required for β-cells to secrete more insulin as a compensatory mechanism specifically under conditions of an insulin resistance state. This is consistent with the observed increase in insulin levels in β-cell specific Y1 knockout mice fed a high-fat diet (Supplementary Fig. 2a). To further functionally test this we performed glucose-stimulated insulin release (GSIS) experiments on isolated islets of C57BL/6JAusb (wild type, WT) and β-cell specific Y1 knockout mice. As shown in Fig. 1c, PYY significantly reduced insulin release from cultured islets stimulated with 11 mM glucose. Importantly, addition of the selective Y1 receptor antagonist BIBO3304^30^ completely abolished PYY-induced inhibition of insulin release (Fig. 1c). Consistent with this, lack of Y1 receptors in β-cells renders them more responsive to glucose with significantly elevated insulin levels already observed with stimulation of 2 mM glucose (Supplementary Fig. 2b). To assess the dynamics of glucose-stimulated insulin secretion in response to PYY and Y1 receptor antagonism on islets we performed islet perifusion studies in WT islets. As shown in Fig. 1d, e, PYY significantly inhibits the first phase response of glucose-stimulated insulin secretion whereas Y1 antagonism employing BIBO3304 treatment resulted in enhanced insulin secretion primarily by increasing the second phase of the insulin secretory response (i.e. 40 min time point). Together, these data demonstrate the critical role of Y1 receptor signaling as a negative regulator of insulin release in pancreatic β-cells under a physiological glucose load.

### Enhanced islet transplantation efficiency employing Y1 deficienct islets

We next conducted a series of studies exploring the potential beneficial clinical effects of enhancing insulin secretion in situations of limited β-cell mass, through manipulation of the Y1 receptor system. We performed islet transplantation experiments and specifically investigated whether Y1 receptor-deficient islets could provide better metabolic control when compared to control WT islets. For this, alloxan-induced diabetic C57BL/6JAusb mice were transplanted with either a minimal amount of 60 islets originating either from WT or Y1^−/−^ mice, respectively, or as a control for islet quality, with a sufficient (optimal) mass of 300 WT islets. Islets of comparable size were hand-picked and as shown in Supplementary Fig. 2c β-cell mass was not significantly different between Y1^−/−^ or WT mice, therefore material with equal insulin content was transplanted. Blood glucose levels were then monitored for the next 20 days. As expected, mice receiving the optimal islet mass showed immediate restoration of blood glucose control (Fig. 1f), whereas mice receiving a minimal number of WT islets had only a slight trend to improved blood glucose control by day 15 post-transplantation, which never completely normalized (Fig. 1f, g). In marked contrast, mice transplanted with the minimal number of 60 Y1^−/−^ islets showed significant glycemic improvement by day 5 post-transplantation and by day 7 blood glucose levels were comparable with the mice receiving the optimal islet mass transplant (Fig. 1f). Consistent with the improved glycemic control, glucose tolerance at day 5 post-transplantation was also significantly improved in mice that received the Y1^−/−^ islets (Fig. 1h, i), which was due to the improved *in vivo* insulin secretory response of the Y1^−/−^ islets (Fig. 1j, k). Improved in vivo insulin secretion was also highlighted by the superior insulin disposition index (DIo) of mice receiving Y1^−/−^ islets; being 2.58 fold higher compared to mice receiving WT islets (DIo = 0.021 ± 0.0017 vs. 0.054 ± 0.019 for Y1^−/−^ vs. WT, respectively; *n* = 4 mice per group). Together, these data demonstrate that reduced Y1 receptor signaling in islets is advantageous for the enhancement of insulin release, and this can have direct relevance to the clinical scenario of islet transplantation where donor islets are of limited supply.

### Inhibition of Y1 receptor signaling improves islet transplantat outcomes

**Fig. 2:**
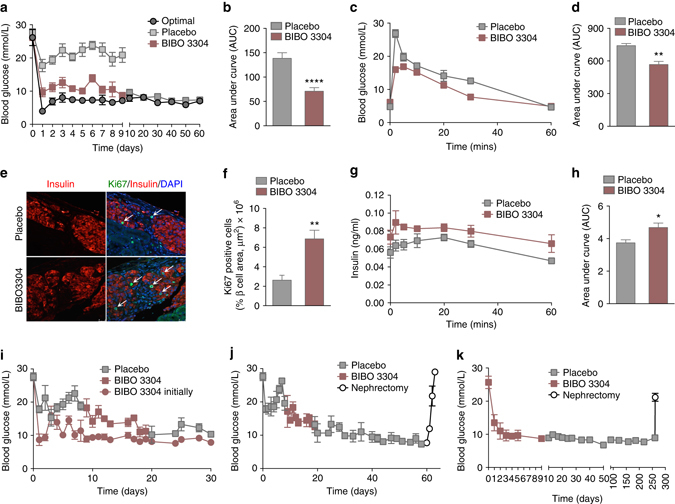
Improved glycemic control in diabetic mice transplanted with WT islets treated with a Y1 receptor antagonist. **a** Alloxan-induced diabetic mice were transplanted with an optimal number of WT islets (300) (*n* = 6) or 60 WT islets (*n* = 12) and treated daily with either 0.5 μM BIBO3304 (*n* = 6) or placebo (*n* = 6) for 9 days, respectively, after which BIBO3304 treatment was stopped but blood glucose levels continuously monitored till day 60 post-transplant. **b** Results for the first 9 days expressed as area under the curve. **c**, **d**, **g**, **h** Diabetic mice were transplanted with 60 WT islets and subsequently orally treated with 0.5 μM BIBO3304 or placebo and i.v. glucose tolerance tests (1 g/kg body weight) (*n* = 4 per group) were performed at day 5 post-transplant. Blood glucose levels and insulin production were monitored. Results are also expressed as area under the curve. **e** Representative photomicrographs of islet transplant grafts from placebo and BIBO3304-treated mice showing immunofluorescent staining for insulin (*red*), Ki67 (*green*) and nucleus counterstained with DAPI (*blue*). *Arrows* indicate Ki67-positive β-cells. **f** Quantification of Ki67-positive β-cells in grafts of placebo or BIBO3304-treated mice (*n* = 3). **i**, **j** Diabetic mice were transplanted with 60 WT islets (*n* = 10 per group) and half of the mice were treated with 0.5 μM BIBO3304 from day 1, and the other half were treated with placebo. Mice originally receiving placebo were then treated with 0.5 μM BIBO3304 from day 9 for 10 days, after which treatment was discontinued. Blood glucose levels were monitored till day 60 after which survival nephrectomy was performed. **k** Diabetic mice were transplanted with 60 WT islets (*n* = 10) and treated with 0.5 μM BIBO3304 for 9 days after which treatment was discontinued. Blood glucose levels were monitored till day 260 after which survival nephrectomy was performed. Data are shown as mean ± s.e.m. *P < 0.05, **P < 0.01, calculated by *t*-test (**b**, **d**, **f**, **h**) or two-way ANOVA analysis

To prove whether these beneficial effects of lack of Y1 receptor signaling could also be induced pharmacologically, we first examined the impact of BIBO3304, a selective non-brain penetrable Y1 receptor antagonist, on glucose induced insulin secretion in vivo. C57BL/6JAusb mice were treated 2 h prior to a bolus glucose load orally with 0.5 μM BIBO3304, a dose known to have physiological action^31^, and serum insulin levels were assessed over the next 60 min. Mice that received BIBO3304 exhibited significantly increased serum insulin levels (Fig. 1l, m) confirming that blocking Y1 receptor signaling is able to enhance physiologically triggered insulin secretion. To further test whether this pharmacological intervention could improve the outcome of islet transplantation, we repeated the transplantation experiments using a minimal number of WT islets, and then treated half of the mouse cohort orally with BIBO3304 and the other half with placebo. As a control for islet quality, a group of mice were transplanted with an optimal islet mass. Recipient mice that were treated with placebo failed to achieve normoglycemia and remained diabetic throughout the course of the experiment (Fig. 2a, b). In contrast, mice transplanted with the minimal amount of WT islets and treated with BIBO3304 rapidly achieved normoglycemia (Fig. 2a). Strikingly, when antagonist treatment was stopped at day 10 post-transplantation these mice were able to maintain normoglycemia till the end of the experiment at day 60 (Fig. 2a). This suggests that transient inhibition of Y1 receptor signaling in the early period after islet transplantation is sufficient to normalize glucose homeostasis. Consistent with the improved glycemic control, glucose tolerance at day 5 post-transplantation was also significantly improved in the BIBO3304-treated group compared to the placebo group (Fig. 2c, d). Consistently, BIBO3304-treated mice also showed a superior DIo being 12.28-fold higher than placebo-treated mice (DIo = 0.066 ± 0.0082 vs. 0.819 ± 0.324 for placebo vs. BIBO3304, respectively; *n* = 4 mice per group). Analysis of BIBO3304-treated compared to untreated islet grafts did not reveal any obvious differences in islet morphology, apoptosis, graft vascularization, or endoplasmic reticulum (ER) stress response (Supplementary Figs. 3, 4a), however, the number of Ki67-positive β-cells in the BIBO3304-treated grafts was significantly increased (Fig. 2e, f). Together, these data demonstrate that the improved glycemic control in BIBO3304-treated mice was a consequence of increased insulin secretion and islet proliferative capacity in response to changes in physiological glucose levels (Fig. 2e–h).

In order to test the time dependence of Y1 receptor antagonism on islet transplantation outcomes, mice were transplanted with a minimal islet mass and half of the cohort were treated daily with BIBO3304 from day 1 and the other half of the cohort were treated with placebo. After 8 days the placebo-treated group also received BIBO3304 treatment. As seen before, mice that initially received placebo treatment were not able to achieve normal glucose levels (Fig. 2i): however, when switched over to BIBO3304 treatment at day 9, these mice showed a significant improvement in glycemic control (Fig. 2i), which was evident within the first few days post BIBO3304 treatment and by day 18 these mice achieved comparable glycemic control to mice that had been fed BIBO3304 from day 1 (Fig. 2i). Significantly, the improvement in glycemic control was maintained even after the subsequent discontinuation of BIBO3304 treatment at day 20 (Fig. 2i). To demonstrate that glycemic control was dependent on the islet grafts a cohort of these mice underwent survival nephrectomy at day 60, resulting in all mice having rapid reversal of glycemic control and a return to hyperglycemia (Fig. 2j). These data indicate that even delayed short-term Y1 receptor intervention can restore normoglycemia in previously non-functioning islet grafts. To test the long-term stability of the BIBO3304 effect upon islet graft function, an additional cohort of mice were transplanted with a minimal islet mass and treated with BIBO3304 for the first 10 days post transplant at which time treatment was discontinued. These mice exhibited stable normoglycemia for > 260 days. After survival nephrectomy all mice rapidly lost glycemic control and returned to hypergylcemia (Fig. 2k) demonstrating that short-term BIBO3304 treatment can provide stable, long-term improvement in islet graft function.

### Y1 antagonism enhances human islet function and transplantat outcomes

**Fig. 3:**
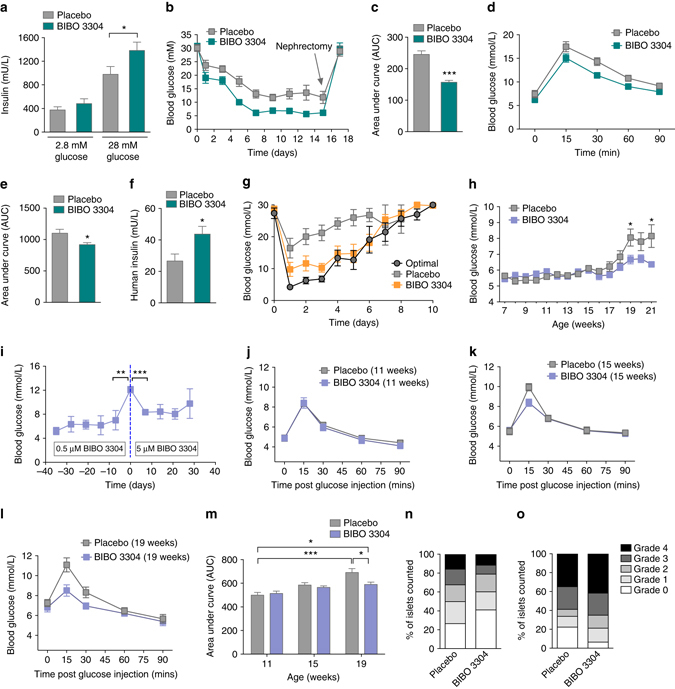
Improved human islet transplant outcomes due to Y1 receptor antagonism. **a** Human islets from three independent donors were isolated and cultured for 48 h. Insulin secretion from human islets was determined in response to 2.8 and 28 mM glucose with or without 1 μM BIBO3304 (*n* = 3; triplicate; three independent donors). **b**, **c** Alloxan-induced NOD RAG1^−/−^ diabetic mice were transplanted with human islets (IEQ 1400) and subsequently orally treated with or without 0.5 μM BIBO3304 and blood glucose levels were monitored for 15 days after which survival nephrectomy was performed (*n* = 7 per group; two independent donors). Results are also expressed as area under the curve. **d**, **e** Human islet recipient mice treated with placebo or 0.5 μM BIBO3304 for 7 days (*n* = 4) were fasted for 6 h and i.p. glucose tolerance tests (1 g/kg body weight) were performed. Results were also expressed as area under the curve. **f** Serum insulin levels in human islet recipient mice were determined at day 15 post-transplantation (*n* = 7). **g** Alloxan-induced diabetic mice were transplanted with a full MHC mismatch (BALB/c islet  −> C57BL/6) optimal number of allogeneic islets (300) (*n* = 6) or 60 allogeneic islets (*n* = 14) and treated daily with either 0.5 μM BIBO3304 (*n* = 6) or placebo (n = 8). **h** 6-week-old female NOD mice were treated with placebo or 0.5 μM BIBO3304 daily and glucose levels monitored weekly (*n* = 16 per group). **i** When the blood glucose levels in BIBO3304-treated mice eventually also reached 12 mM, a 10-fold higher dose of BIBO3304 was given in an attempt to reverse the hyperglycemia (*n* = 3). **j**, **k**, **l** Glucose tolerance tests were performed in BIBO3304-treated and placebo-treated NOD mice at 11, 15, and 19 weeks of age, respectively (*n* = 6 per group). **m** Results were also expressed as area under the curve. **n**, **o** Insulitis was scored on islets from NOD mice either treated with 0.5 μM BIBO3304 or placebo at 12 or 22 weeks of age (*n* = 6, *n* = 5), respectively. Data are means ± s.e.m. *P < 0.05, **P < 0.01; ***P < 0.001 calculated by *t*-test (**a**, **c**, **e**, **f**) or two-way ANOVA analysis

We next investigated the physiological relevance of Y1 receptor signaling on insulin secretion in human islets. To investigate whether and which Y-receptors are present in human islets, we performed micro-array analysis and confirmed that consistent with mouse β-cells, the Y1 receptor is the only Y-receptor expressed at significant levels by human islets (Supplementary Table 1). In order to test the physiological relevance of Y1 receptor signaling in human islets, human islets were cultured and stimulated with glucose in the presence or absence of the Y1 receptor antagonist BIBO3304. Similar to rodent islets, human islets treated with BIBO3304 exhibited enhanced insulin secretion in response to a glucose challenge (Fig. 3a). We next determined whether pharmacological inhibition of Y1 receptor signaling with BIBO3304 would improve human islet function *in vivo* after islet transplantation. For this, human islets were transplanted into diabetic NOD RAG1^−/−^ mice that were subsequently treated with BIBO3304 or placebo after transplant and their blood glucose levels were monitored. Placebo-treated mice did not show a return to normoglycaemic levels (i.e., < 10 mM glucose) over the monitoring period of 15 days (Fig. 3b, c). In contrast, mice treated with BIBO3304 showed significantly accelerated recovery of glucose control, reaching normal glucose levels by day 5. Consistent with the improved glycemic control, glucose tolerance was also significantly improved in the BIBO3304-treated group compared to the placebo group (Fig. 3d, e). This improved glycemic control in BIBO3304-treated mice was accompanied by increasedcirculating human insulin levels (Fig. 3f). Together these data demonstrate that the action of Y1 receptor signaling to modulate insulin secretion is conserved in human islet physiology.

To assess Y1 receptor antagonism as a potential treatment option in islet transplant patients a model that more closely mimics the human situation was chosen. For this, islets were transplanted across a full MHC mismatch (BALB/c islet  −> C57BL/6 diabetic recipient) to mimic the situation of human islet transplantation with immune-mediated allogeneic rejection. Under this setting, the transplantation of the optimal 300 islets restored metabolic control whereas the minimal mass of 60 islets in mice treated with placebo did not (Fig. 3g). Importantly, the administration of Y1 receptor antagonist rescued the function of the limiting islet mass such that mice receiving 60 islets and treated with BIBO3304 show complete restoration of full metabolic function equivalent to mice that received 300 islets (Fig. 3g). These experiments demonstrate that Y1 receptor antagonism is able to rescue islet function even in the context of ongoing immune-mediated allograft rejection. Of note, the time to rejection for mice receiving 60 islets plus Y1 receptor antagonist or 300 islets was the same, suggesting BIBO3304 was not impacting on the immune response but rather enhancing islet function, consistent with our earlier results.

### Y1 antagonism improves glycemic control and delays the onset of type 1 diabetes

Due to the success of BIBO3304 in controlling blood glucose in mice transplanted with a minimal number of mouse or human islets, we considered whether this would also be effective in other settings with reduced insulin secretory capacity. We therefore next determined whether Y1 receptor antagonism could extend the period of normoglycemia in autoimmune prone non-obese diabetic (NOD) mice. Abnormalities of β-cell function can be detected in NOD mice before they develop diabetes^32^ and this is also true in humans who have dysglycemia before they develop T1D^33^. Similarly, following diagnosis of T1D there is a period with reduced β-cell function that sometimes is still sufficiently effective for insulin treatment to be ceased for a period of time is called the ‘honeymoon period’. We tested if BIBO3304 would improve glycemic control in NOD mice prior to diabetes with potential application to reducing dysglycemia before and after diagnosis of diabetes and possibly prolonging the time to diagnosis of T1D and duration of the honeymoon period.

In order to test the responsiveness of NOD mice to Y1 receptor antagonism we treated 7-week-old NOD mice with a single dose of 0.5 μM BIBO3304 for 1 h before the onset of the dark phase (1900 hours) and measured glucose levels at 0900 hours the following day. Interestingly, NOD mice treated with BIBO3304 displayed a significant decrease in glucose levels (Placebo, 5.35 ± 0.11 mM; BIBO3304, 5.03 ± 0.09 mM; *P < 0.05), which is associated with a strong trend to increased insulin levels (Placebo, 73.31 ± 9.42 pM; BIBO3304, 103.2 ± 11.87 pM; *P* = 0.06), indicating that β-cells in a T1D environment are responsive to the inhibition of Y1 receptor signaling. To determine whether BIBO3304 treatment would prevent or delay the onset of diabetes in NOD mice, we treated a cohort of NOD mice daily with either BIBO3304 or placebo from 6 weeks of age onwards. As expected placebo-treated NOD mice exhibited worsening glucose control over time and eventually developed overt diabetes (Fig. 3h). In contrast, BIBO3304-treated NOD mice showed a marked resistance to the onset of hyperglycemia for an extended period (2–4 weeks) (Fig. 3h), but eventually these mice treated with 0.5 μM BIBO3304 also progressed to hyperglycemia (Fig. 3i). However, when these BIBO3304-treated NOD mice were diagnosed with hyperglycemia (glucose levels >12 mM), increasing the BIBO3304 dose 10-fold was able to reverse this trend and further delay the onset of overt hyperglycemia (Fig. 3i). Consistent with the concept that BIBO3304 treatment improves β-cell function, NOD mice treated with BIBO3304 showed significantly better glucose tolerance over the time course of BIBO3304 treatment at 15 and 19 weeks of age, respectively (Fig. 3j–m). In comparison, placebo-treated NOD mice showed a worsening glucose tolerance consistent with their progression to overt hyperglycemia and diabetes (Fig. 3j–m). The improvement in glycemic control provided by BIBO3304 at least in the early phase (12 weeks of age) was independent of effects on the immune system as there were no differences in T-cell and B-cell populations in placebo vs. BIBO3304-treated mice. In particular there was no evidence of a change in the speed of an autoimmune attack as there was no difference in critical immune markers associated with autoimmune diabetes in the spleen, pancreatic lymph nodes, and mesenteric lymph nodes (Supplementary Fig. 5). Furthermore, BIBO3304 treatment had no significant effect on the expression levels of classical ER stress-mediating gene-products in islets from NOD mice excluding this pathway as a likely reason for the observed improved islet function (Supplementary Fig. 6a). Investigating the degree of immune destruction in these NOD mice, insulitis scores revealed that overall there was no significant difference between placebo or BIBO3304-treated animals at 12 weeks of age (Fig. 3n) and again further into disease progression at 22 weeks of age (Fig. 3o), or β-cell area (Supplementary Fig. 6b). This data suggests that improving insulin secretion due to Y1 receptor antagonism is the main driver of the prolonged control of glucose homeostasis in the NOD mice. Together, these results suggest Y1 receptor antagonism may be beneficial clinically to prolong the honeymoon period in new-onset T1D patients by enhancing insulin secretion.

**Fig. 4:**
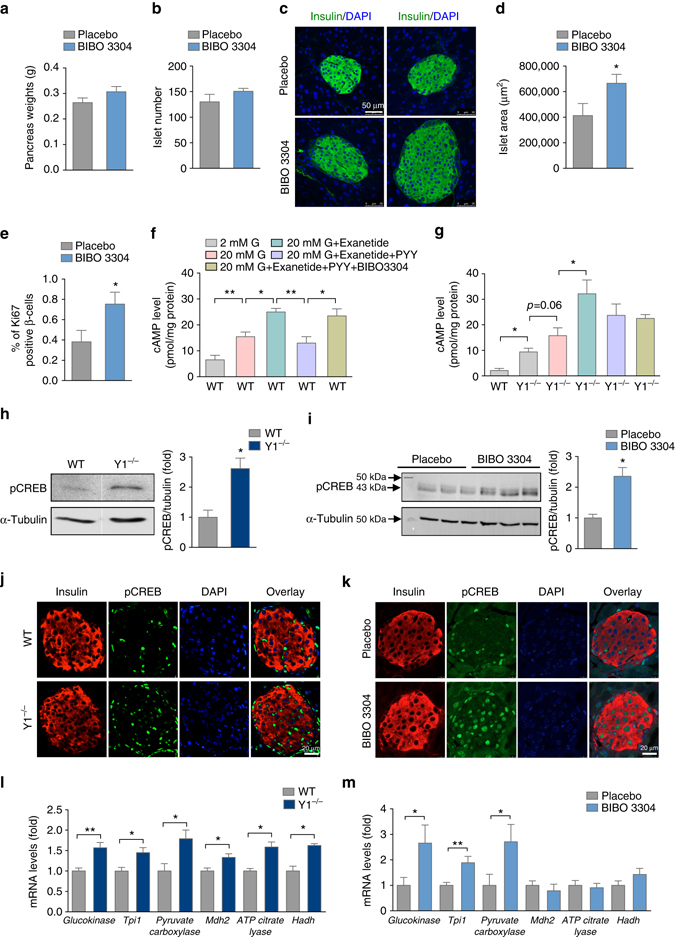
Y1 receptor signaling regulates β-cell function via a cAMP-CREB-dependent pathway. **a** Pancreas weight of BIBO3304 vs. placebo-treated WT mice (*n* = 3). **b** Total islet number of BIBO3304 compared to placebo-treated WT mice (*n* = 3). **c** Representative photomicrographs of islets stained for insulin from BIBO3304 and placebo-treated WT mice (*n* = 3). **d** Total islet area of BIBO3304 compared to placebo-treated WT mice (*n* = 3). **e** Quantification of Ki67-positive β-cells in islets from BIBO3304 or placebo-treated WT mice. **f** cAMP production of WT islets in response to 2 and 20 mM glucose co-stimulation with GLP-1 receptor agonist, Exenatide (100 nM), in the presence or absence of PYY (50 nM), or BIBO3304 (1 μM) (*n* = 5). **g** cAMP production of Y1^−/−^ islets in response to 2 and 20 mM glucose co-stimulation with GLP-1 receptor agonist, Exenatide (100 nM), in the presence or absence of PYY (50 nM), or BIBO3304 (1 μM) (*n* = 5). **h**, **i** Pancreatic islets from WT mice treated with placebo or BIBO3304 and Y1^−/−^ mice were isolated and subjected to SDS–PAGE and western blot analysis using anti-p-CREB and α-tubulin antibodies (*n* = 5–7). **j**, **k** Representative fluorescence micrographs of pancreata from WT mice treated with placebo or BIBO3304 and Y1^−/−^ mice showing staining for insulin (*red*), phospho-CREB (*green*) and nuclear counterstained with DAPI (*blue*), *scale bar* = 20 μm. **l**, **m** Pancreatic islets from Y1^−/−^ and WT mice treated with placebo or BIBO3304 were isolated and genes involved in insulin secretory pathways including *glucokinase, Tpi1, pyruvate carboxylase, Mdh2, ATP citrate Lyase*, and *Hadh* were determined using quantitative RT-PCR. *Cyclophillin A* and *Rpl19* were used as a housekeeping gene (*n* = 3). Data are means ± s.e.m. *P < 0.05, **P < 0.01, calculated by *t*-test (**c**, **e**, **f**) or two-way ANOVA analysis

To investigate the general effects chronic antagonism of Y1 receptor signaling has on islets, we treated a set of 7-week-old WT mice with BIBO3304 or placebo for 7 weeks, upon which animals were sacrificed, pancreata isolated, and analyzed. While showing a slight trend, there was no significant increase in pancreas weight in the BIBO3304-treated mice (Fig. 4a) nor any difference in total islet numbers (Fig. 4b). Interestingly, however, islets from BIBO3304-treated mice appeared to be larger in size (Fig. 4c) and total islet area due to an increase of individual islet size was significantly greater in the BIBO3304-treated mice compared to the placebo group (Fig. 4d). This increased islet size was most likely due to increased β-cell proliferation consistent with the increased number of Ki67-positive cells seen in the BIBO3304 group (Fig. 4e). Taken together, this suggests that Y1 receptor antagonism in the β-cells may not only promote insulin secretion but also enhance β-cell proliferation.

### Y1 receptor signaling regulates β-cell function via a cAMP dependent pathway

In order to gain mechanistic insight on the consequences of Y1 receptor signaling in islet cells we performed a series of in vitro experiments. Since the Y1 receptor interacts with G_i/o_ G-proteins, which act to inhibit adenylate cyclase activity and cAMP levels modulate insulin secretion, we investigated cAMP production in control and Y1^−/−^ islets in response to glucose. For this we tested whether pharmacologically blocking Y1 receptor signaling in islets can reproduce this effect ex vivo. To mimic physiological conditions we treated islets isolated from WT mice with the GLP-1 receptor agonist ‘Exenatide’ in the presence of glucose to raise cAMP levels. As shown in Fig. 4f, cAMP was significantly upregulated in the presence of 100 nM Exenatide and 20 mM glucose compared to glucose alone. The addition of 50 nM PYY was able to significantly reduce the Exenatide-induced increase in cAMP and this inhibitory effect was abolished in the presence of the Y1 receptor specific antagonist BIBO3304 (Fig. 4f). To investigate whether the GLP-1-induced increase in cAMP could be further enhanced in the absence of Y1 receptor signaling, we also performed the experiment on islets derived from Y1^−/−^ mice. The addition of Exenatide resulted in a further increase in cAMP levels in Y1^−/−^ islets pretreated with 20 mM glucose (Fig. 4g). Importantly, the addition of PYY or PYY and BIBO3304 had no significant effect on reducing cAMP levels in the Y1^−/−^ islets consistent with a Y1 receptor-dependent effect.

Taken together, these data demonstrate that Y1 receptor signaling can counteract the Exenatide-induced enhancement of insulin secretion via direct effects on cAMP production. Further support for this concept comes from studies where we could show that protein levels of phosphorylated cAMP responsive element-binding protein (pCREB) are significantly upregulated in islets of Y1^−/−^ or BIBO3304-treated WT mice as determined by western blotting (Fig. 4h, i) and in situ immunofluorescence (Fig. 4j, k).

Since CREB expression may also be influenced by Ca^2+^ and Ca^2+^ is a vital part of the exocytosis of insulin containing secretory granules, we performed [Ca^2+^]_i_ measurements to investigate whether Y1 receptor signaling is critical for these events. A repetitive stimulation with 11 mM glucose induced repeated [Ca^2+^]_i_ increases in mouse single β-cells as expected and PYY (10 nM), added to the superfusion solution 5 min prior to the second glucose stimulation, did not affect [Ca^2+^]_i_ responses to 11 mM glucose (Supplementary Fig. 7a). The ratio of the peak amplitude of the [Ca^2+^]_i_ response to the second glucose stimulation (S2) compared to that of the first stimulation (S1), and the S2/S1 ratio was also unaltered by PYY (Supplementary Fig. 7b). Furthermore, PYY did not affect the oscillation of [Ca^2+^]_i_ during the second phase response to 11 mM glucose (Supplementary Fig. 7c) nor did it influence the KCl (25 mM)-induced [Ca^2+^]_i_ increases at 2 mM glucose in β-cells (Supplementary Fig. 7d, e). As such, Ca^2+^ does not seem to play a major role in PYY/Y1 receptor mediated downstream processes in β-cells suggesting that Y1 receptor signaling primarily inhibits the CREB-dependent pathway to influence insulin secretion.

We next investigated whether key cellular molecules known to be involved in the insulin secretion process are under the control of the CREB transcription factor. The expression of several genes involved in glycolysis such as the rate-limiting enzyme glucokinase^34^, the anaplerotic enzyme pyruvate carboxylase (*Pc*)^35^ and in mitochondrial oxidation process, malate dehydrogenase (*Mdh2*)^36^, ATP citrate lyase and 3-hydroxyacyl-CoA dehydrogenase (*Hadh*)^37^ are known to respond to CREB. Consistent with this, quantitative real-time PCR (qPCR) performed on RNA isolated from Y1^−/−^ islets confirmed an increase in mRNA expression of these critical metabolic genes in the absence of Y1 receptor signaling (Fig. 4l). Absence of Y1 receptor signaling also increased the expression of CREB downstream target genes controlling mitochondrial oxidation including *Mdh2*, ATP citrate lyase, and *Hadh* (Fig. 4l). Consistent with this notion that Y1 receptor signaling via the cAMP/CREB pathway controls insulin secretion, repeating this analysis in WT islets that were treated with BIBO3304 revealed that while not all the genes investigated were upregulated a similar pattern of altered gene expression of glycolysis controlling genes was observed (Fig. 4m). It is not too surprising that the effects of Y1 receptor deletion, which occurred in the germline, may result in a more extended change in expression of β-cell specific genes than what could be expected from a short-term treatment regime using an antagonist. Interestingly, however, the effect that BIBO3304 has on the expression of the set of glycolysis controlling genes that are increased seems to be more pronounced and almost double suggesting modulating this set of genes is sufficient to produce the increase in insulin secretion.

In contrast to changes in these key metabolic genes, genes that influence the maintenance of the β-cell phenotype were not significantly altered (Supplementary Fig. 8a–c). Entry of glucose into the β-cell with subsequent glycolysis and mitochondrial oxidation is a critical trigger for the release of appropriate amounts of insulin in response to changes in physiological glucose. However, expression levels of *Glut2*, the key transporter governing glucose uptake into the β-cell and subsequent entry into glycolysis was not significantly changed in the β-cells of Y1^−/−^ mice (Supplementary Fig. 8d), again suggesting that Y1 receptor signaling predominantly regulates cAMP levels and CREB activity, which via upregulation of enzymes that are central in controlling glycolysis plays a key role in the physiological control of insulin secretion.

## Discussion

By using genetic and pharmacological approaches our results reveal that Y1 receptors play an essential and conserved role in β-cell physiology by providing an inhibitory action in modulating insulin release in response to a glucose load. This finding also highlights a potential causal role for impaired Y1 receptor signaling in the development of metabolic diseases such as obesity and hyperinsulinemia. Importantly, we demonstrate that manipulating the Y1 receptor system via pharmacological intervention may be a useful therapeutic approach to boost β-cell function under conditions where insulin secretion is limited. Our findings have the potential for a direct clinical application in the context of pancreatic islet transplantation, which is emerging as an important therapy to treat T1D and hypoglycemic unawareness^5^ but is limited in its scope by the poor function of the transplanted islets after engraftment and scarcity of donor material. Indeed, with current best practice most islet transplant recipients require 2–3 islet transplants to achieve normal metabolic control with insulin independence^38^. Based on our studies here, by employing a Y1 antagonist as co-treatment with islet transplantation, the required number of islets to achieve the same level of insulin production could be significantly reduced thereby reducing the number of donors required per recipient and consequently increasing the number of patients who could benefit from islet transplantation. In addition to the potential for improving the current clinical practice of adult islet transplantation, Y1 receptor antagonism may also be applicable to newly emerging therapies of islet transplantation that take advantage of alternative islet sources, such as porcine (xenogeneic) or stem cell-derived (both stem cell and induced pluripotent stem (iPS) cell) islets. As the effect of Y1 receptor antagonism is highly effective in the immediate period after transplant and can be suspended after a short treatment period, targeting the Y1 receptor brings the additional advantage of requiring only a temporary treatment regime.

## Methods

### Mice

All animal care and experiments were approved by the Garvan/St. Vincent’s Animal Ethics Committee. Y1^lox/lox^ mice were generated as previously described^39^ and crossed with mice expressing the Cre recombinase gene under the control of the rat insulin-2 promoter Tg(Ins2-cre)25Mgn/J(INS2^cre/+^)^40^ to generate Y1^lox/lox^/INS2^cre/+^ mice. INS2^cre/+^ mice were also crossed onto *Gt(ROSA)26Sor* ^*tm9(EGFP/Rpl10a)Amc*^/J mice (JAX stock# 024750) in order to produce β-cell specific expression of the EGFP–L10a fusion protein for mRNA isolation and qPCR analysis. For clarity, islet tissue from these mice is referred to as WT and Y1^−/−^ respectively, throughout the text. Age-matched and sex-matched mice on a C57BL/6Ausb background were used for all experiments except stated otherwise. Female NOD mice were purchased from Australian BioResources Pty Ltd (ABR, Moss Vale, NSW, Australia). Mice were housed under a controlled temperature of 22 °C and a 12-hour light cycle (lights on from 0700 to 1900 hours) with *ad libitum* access to water and a standard chow diet (6% calories from fat, 21% calories from protein, 71% calories from carbohydrate, 14.0 MJ/kg, Gordon’s Specialty Feeds, Australia). To avoid the stress caused by gavage, specific Y1 receptor antagonist BIBO3304 (Tocris Bioscience) was dissolved in distilled water and administrated daily in a form of jelly for the duration stated in the text. This method of drug delivery was developed in our lab and described previously^31^. In brief, we trained mice to voluntarily eat a vehicle jelly before the start of an experiment. After 2–5 days training, over 95% of mice consumed the entire portion of jelly (195 μl for a 25 g mouse) within 1 min of being placed in the cage and maintained a high avidity for jelly throughout the study period. At the commencement of an experiment, mice received BIBO3304 containing jelly once per day for the time period indicated in each study, while control mice received vehicle jelly.

### RNA extraction and quantitative RT-PCR

Pancreatic islets were isolated and immediately frozen in liquid nitrogen, and RNA was extracted using Trizol Reagent (Sigma, St. Louis, MO). Isolated mRNA was reverse transcribed into cDNA using Superscript III First-Strand Synthesis System (Invitrogen, Australia) and processed for qPCR with the Light-Cycler 480 Real-Time PCR system (Roche, Switzerland) for *Gk, Tpi1,Acly, Pc, Mdh2, Hadh, Glut2, Pdx1, MafA, Nkx6.1, NeuroD1* as indicated in the Supplementary Table 2 and normalized with a housekeeping gene Ribosomal protein L19 (*Rpl-19)* (5′-CTCGTTGCCGGAAAAACA-3′ and 5′-TCATCCAGGTCACCTTCTCA-3′). *Npy1r* (Y1 receptor) gene (5′-CACAGGCTGTCTTACACG-3′ and 5′-GCGAATGTATATCTTGAAGTAG-3′). The amplification condition used in all the RT-qPCR experiment was: 94 °C for 30 s, 60 °C for 30 s, 72 °C for 20 s for 40 cycles.

### Glucose-stimulated insulin secretion ex vivo

Pancreas from 8-week-old to 10-week-old WT and Y1^−/−^ mice were perfused with cold liberase (Roche) solution via the common bile duct and subsequently pancreata were removed and incubated at 37 °C for 16 min. The digestion was stopped by addition of Krebs-Ringer Bicarbonate buffer containing 10% newborn calf serum followed by mechanical disruption of the pancreas and washing of the islets. Islets were further separated from other pancreatic tissue using a Ficoll-Paque PLUS (GE Healthcare) density gradient and hand-picked under a stereomicroscope. Islets were subsequently incubated in RPMI-1640 medium (containing 10% fetal calf serum, penicillin and streptomycin, HEPES, 11 mM glucose, and 2 mM glutamine) in a 37 °C, 5% CO_2_ incubator for 48 h. GSIS assays were performed as previously described^41^. Briefly, islets were pre-incubated for 1 h in HEPES-buffered KRB containing 0.1% BSA and 2 mM glucose. Experiments were performed in triplicate with batches of five islets incubated at 37 °C, 5% CO_2_ for 1 h in 130 μl KRB containing 0.1% BSA, supplemented with 2 mM, 11 mM, or 20 mM glucose or 2 mM glucose with KCl (25 mM) in with or without 100 nM Exenatide (GLP-1 receptor agonist, Byetta) and/or 50 nM PYY and/or 0.5 μM BIBO3304 as indicated in the text. Insulin release was determined by insulin RIA kit (Linco Research, St. Charles, MO, USA). Insulin DIo was calculated from the formula ∆*I*_0_−*I*_30_/∆*G*_0_−*G*_30_ × 1/fasting insulin; where 1/fasting insulin=serum insulin level determined by ELISA (pM) and G=blood glucose (mmol/l).

### Glucose tolerance tests and in vivo GSIS

Glucose tolerance tests (GTT) were performed on 6 h-fasted mice that were administered i.p. with glucose (1 g/kg body weight). Blood glucose levels were assessed at 0, 15, 30, 60, and 90 min after glucose administration using a Accu-chek Go glucometer (Roche, Dee Why, Australia). Serum during GTT was collected and stored for subsequent insulin assays using insulin RIA kits (Linco Research). Fed and fasted serum insulin levels were also measured using insulin RIA kits (Linco Research). For islet transplantation, mice were fasted overnight and i.v. administered with glucose (1 g/kg body weight) and blood glucose levels were assessed at 0, 2, 5, 10, 15, 30, and 60 min after glucose administration using a glucometer. Blood samples during GTT were collected and stored for subsequent insulin assays using an ultra-sensitive commercially available ELISA kit (Crystal Chem, Downers Grove, USA). Human insulin levels during human islet transplantation were determined using human insulin ELISA kit (Mercodia AB, Sweden).

### cAMP measurement assays

Isolated islets were incubated in RPMI-1640 medium (containing 10% fetal calf serum and penicillin and streptomycin, HEPES, 11 mM glucose, and 2 mM glutamine) in a 37 °C, 5% CO_2_ incubator for 24 h. Batches of 40 islets were washed with KRB buffer and subsequently incubated with KRB buffer containing 0.1% BSA and 2 mM glucose at 37 °C, 5% CO_2_ for 1 h before the assay. Islets were cultured with 2 mM, 11 mM, or 20 mM glucose, 100 nM Exenatide (Byetta, AstraZeneca) in the presence of 0.5 mM IBMX (Sigma-Aldrich) and intracellular accumulation of cAMP was measured over a period of 1 h. Islets were lysed in 150 μl 0.1 M HCl for 20 min and cAMP levels were measured using the ‘Direct cAMP ELISA kit’ (Enzo Life Sciences, Farmingdale, NY) according to the manufacturer’s protocol. Final cAMP concentrations were normalized by total protein content.

### Measurements of [Ca^2+^]_i_ in single β-cells

Islets isolated from male C57BL/6Ausb mice were dispersed into single cells in Ca^2+^-free HKRB. The single cells were plated sparsely on coverslips and maintained for 1 day at 37 °C, 5% CO_2_ incubator in Eagle’s minimal essential medium containing 5.6 mM glucose supplemented with 10% fetal bovine serum, 100 μg/ml streptomycin, and 100 U/ml penicillin. Dissociated single β-cells on coverslips were mounted in an open chamber and superfused in HKRB. Cytosolic Ca^2+^ concentrations ([Ca^2+^]_i_) in single β-cells were measured at 33 °C by dual-wavelength fura-2 microfluorometry with excitation at 340/380 nm and emission at 510 nm using a cooled charge-coupled device camera. The ratio image was produced on an Aquacosmos system (Hamamatsu Photonics, Hamamatsu, Japan). Data were taken exclusively from the cells, which fulfilled the reported morphological and physiological criteria of β-cells^42^.

### Immunofluorescent staining on grafts and/or pancreas

Insulin (Sigma-Aldrich; #I2018, produced in mouse, 1:500 dilution), insulin (H-86, Santa Cruz, #9168, produced in rabbit, 1:400 dilution), p-CREB (phospho-CREB-Ser133, Cell Signaling; #9198, 1:500 dilution), CD31 (Abcam; #ab28364, 1:50 dilution), and Ki67 (Ki67 [SP6], Thermo Fisher Scientific; #RM-9106, 1:250 dilution) immunofluorescence was performed on paraformaldehyde-fixed, paraffin-embedded grafts, or pancreatic sections, as described previously^43^. Briefly, grafts or whole pancreas, fixed in 4% PBS-buffered paraformaldehyde overnight at 4 °C before being processed and embedded in paraffin. Sections were cut at 5 μm for pancreas and kidney grafts, deparaffinized, rehydrated, and incubated with a Target Retrieval Solution (pH = 6, DAKO Corp, Carpinteria, CA, USA) in a pressure cooker. Slides were then washed in distilled water and incubated with 5% goat serum in PBS containing 1% BSA for 1 h at room temperature. Subsequently sections were incubated overnight at 4 °C in hydration chambers with the respective primary antibody (1 h at room temperature for CD31). Slides were then washed in PBS and incubated with the respective secondary antibody for 1 h at room temperature. The slides were washed in PBS and cover slipped with ProLong Gold Antifade mounting medium (Thermo Fisher Scientific Inc. Waltham, MA, USA). Sections were imaged via a Leica SP8 confocal microscope. Entire sections of the pancreas were scanned and imaged using the Leica LAS Power Mosaic (Leica Microsystems, Wetzlar, Germany). For quantification of Ki67-positive cells in the islet grafts from placebo or BIBO3304-treated mice, six sections separated by at least 150 μm were used for each mouse, *n* = 3 mice per group. Investigators were blinded to outcome assessment.

### Western blotting

Isolated mouse islets were mechanically homogenized in ice cold RIPA lysis buffer (50 mM Hepes [pH 7.4], 1% (v/v) Triton X-100, 1% (v/v) sodium deoxycholate, 0.1% (v/v) SDS, 150 mM NaCl, 10% (v/v) glycerol, 1.5 mM MgCl_2_, 1 mM EGTA, 50 mM NaF, 2 mM phenylmethysulfonyl fluoride, 1 mM sodium vanadate), and clarified by centrifugation (100,000×*g* for 5 min at 4 °C). Cell lysates were resolved by SDS–PAGE and immunoblotted with phospho-specific CREB antibody (phospho-CREB-Ser133, Cell Signaling; #9198, 1:1000 dilution) or alpha-tubulin monoclonal antibody (clone B-5-1-2, #T6074, Sigma-Aldrich, 1:1000 dilution) as a loading control.

### Islet transplantation

To induce diabetes, 8–10-week-old recipient C57BL/6Ausb mice were i.v. injected with alloxan tetrahydrate (Sigma-Aldrich) in injectable grade water (20 mg/ml) at a dose of 110 mg/kg body weight. Mice with blood glucose values ≥18 mmol/l were selected as transplant recipients. Islets were isolated from pancreas of either Y1^−/−^ mice or control donor mice and transplanted into the recipient diabetic mice (syngeneic) as previously described^44^. In brief, on the transplant day, islets were prepared from the pancreata of donor mice and either 300 or 60 handcounted islets were transplanted into individual recipient mice. For the transplant, the kidney was accessed by a left flank incision and brought into the wound by gentle blunt dissection. A small nick was made in the kidney capsule at the inferior renal pole and the islets were deposited through the nick toward the superior pole of the kidney. Blood glucose levels were monitored daily. Transplanted mice underwent different regimes as detailed in the text. Nephrectomy was performed at a specific postoperative day as indicated in the text or figures.

### Treatment plan, monitoring and study endpoints in NOD mice

Seven-week-old female NOD mice were fed with placebo jelly or 0.5 μM BIBO3304 containing jelly daily. When a blood glucose level of > 12 mM was recorded in BIBO3304-treated mice, these mice were given an increased dose (5 μM BIBO3304) to examine the possibility of reverting the trend. In another experiment, female NOD mice were treated daily with either 0.5 μM BIBO3304 containing jelly or vehicle jelly. Glucose levels were monitored weekly prior to 12 weeks of age and twice per week after 12 weeks of age. In these mice a GTT was carried out at 4, 8, and 12 weeks post treatment or daily post hyperglycemia. Animals were also monitored daily for signs of deteriorating health, as indicated by weight loss, slow movement, hunched posture, and polyuria. Mice deemed morbid by the above criteria were sacrificed. Pancreas from 12-week-old or 22-week-old female NOD mice treated with BIBO3304 or placebo were formalin fixed, paraffin embedded, and stained with H&E to evaluate islet insulitis. The histology of the pancreatic islets was evaluated by using a bright field microscopy. The insulitis was scored in four levels. The total number of the islets in the preparations were taken into account together with the infiltration of lymphocytes. Representations of the different insulitis scores were shown as below: Grade 0 = no insulitis, representing normal islet morphology; Grade 1 = peri-islet insulitis, representing < 25% infiltration with inflammation concentrated around the islet; Grade 2 = intermediate insulitis, representing 25–50% of the islet was penetrated by inflammatory infiltrate; Grade 3 = intra-islet insulitis, representing 50–75% of the islet was penetrated by inflammatory infiltrate; and Grade 4 = complete islet insulitis, representing > 75% of the islet was penetrated by inflammatory infiltrate. The treatments were unknown to the scoring person to ensure blind evaluation.

### Human islet isolation, transplantation, and treatment plan

We obtained approval for performing human islet studies from St Vincent’s Institute of Medical Research and St. Vincent’s Clinical School Human Research Ethics Committee. Consent for use of the islets for research was given by relatives of the donors. Isolated human islets were prepared from two independent deceased donors and purified using the modified method as previously described^45, 46^. Human islets were cultured overnight and transplanted of 1400 cultured islet equivalents into alloxan-induced NOD RAG1^−/−^ diabetic mice as described previously^47^. In brief, the kidney was accessed by a left flank small incision and brought into the wound by gentle blunt dissection. A small nick was made in the kidney capsule at the inferior renal pole. The islets were gently placed through the nick toward the superior pole of the kidney. Subsequently, recipient mice were either orally administered with placebo or Y1 receptor antagonist, BIBO3304 (0.5 μM) daily and graft function was determined by monitoring fed blood glucose levels up to 18 days. Glucose tolerance tests (i.p. 1 g/kg body weight) were performed on day 7 post-transplantation. Nephrectomy was performed at postoperative day 18.

### Flow cytometry

Cell suspensions of spleen and lymph nodes were prepared according to standard protocols and stained for FACS analysis in PBS containing 0.5% BSA, 2 mM EDTA, and 0.02% sodium azide using the following Abs (obtained from BD Pharmingen, unless otherwise stated): Pacific-Blue-conjugated anti-CD4, FITC-conjugated, or PerCP-Cy5.5-conjugated (eBioscience) anti-CD8, PerCP-Cy5.5-conjugated anti-B220, allophycocyanin-conjugated anti-CD44, FITC-conjugated, or PerCP-Cy5.5-conjugated (eBioscience) anti-CD62L, PE-conjugated CD25 (PC61.5), H-2K(d) Mouse IGRP 206-214 VYLKTNVFL AlexaFlour 647-Labeled Tetramer (NIH Tetramer Core Facility), I-A(g7) BDC2.5 mimotope AHHPIWARMDA PE-Labeled Tetramer (NIH Tetramer Core Facility). Samples were acquired on a LSRII Sorp (BD Biosciences) and data were analyzed with FlowJo software (Tree Star). Gating strategy: Following labelling events were gated on PI-lymphocytes (to exclude dead cells) using forward and side light scatter parameters, then gated for CD4+CD8−, or CD4−CD8+ events respectively. To assess the frequency of tetramer-positive events gates were drawn by setting the control tetramer gate to 0.1% and using this gate for the corresponding tetramer stained sample. Data is the percentage of tetramer-positive events among all CD4+ or all CD8+ cells, respectively.

### Statistical analyses

All data are expressed as mean ± SEM. A two-tailed Student’s *t*-test was used to test difference between two groups of mice. Differences among groups of mice were assessed by two-way ANOVA or repeated-measures ANOVA. Bonferroni post hoc tests were performed to identify differences among means. *F* test was used to compare variances. Statistical analyses were assessed using Prism software (GraphPad Software 6.0 f, Inc, LaJolla, CA, USA). Sample size was predetermined based on the variability observed in prior experiments and on preliminary data. All experiments requiring the use of animals or animals to derive cells were subject to randomization based on litter. Investigators were not blinded to outcome assessment except were otherwise stated. Differences were regarded as statistically significant if *P < 0.05; **P < 0.01; ***P < 0.001 ****P < 0.0001.

### Data availability

The data that support the findings of this study are available from the corresponding author upon reasonable request.

## Electronic supplementary material

Supplementary Information

Peer Review file
