# Supplementary material for: Inhibition of Y1 receptor signaling improves islet transplant outcome
Source: Nat Commun. 2017 Sep 8;8:490. doi: 10.1038/s41467-017-00624-2 (PMC5591241; doi:10.1038/s41467-017-00624-2)
Supplement: Supplementary file 1 — Supplementary Information [file 41467_2017_624_MOESM1_ESM.pdf]

Description of Supplementary Files

File name: Supplementary Information
Description: Supplementary figures and supplementary tables.

File name: Peer review file

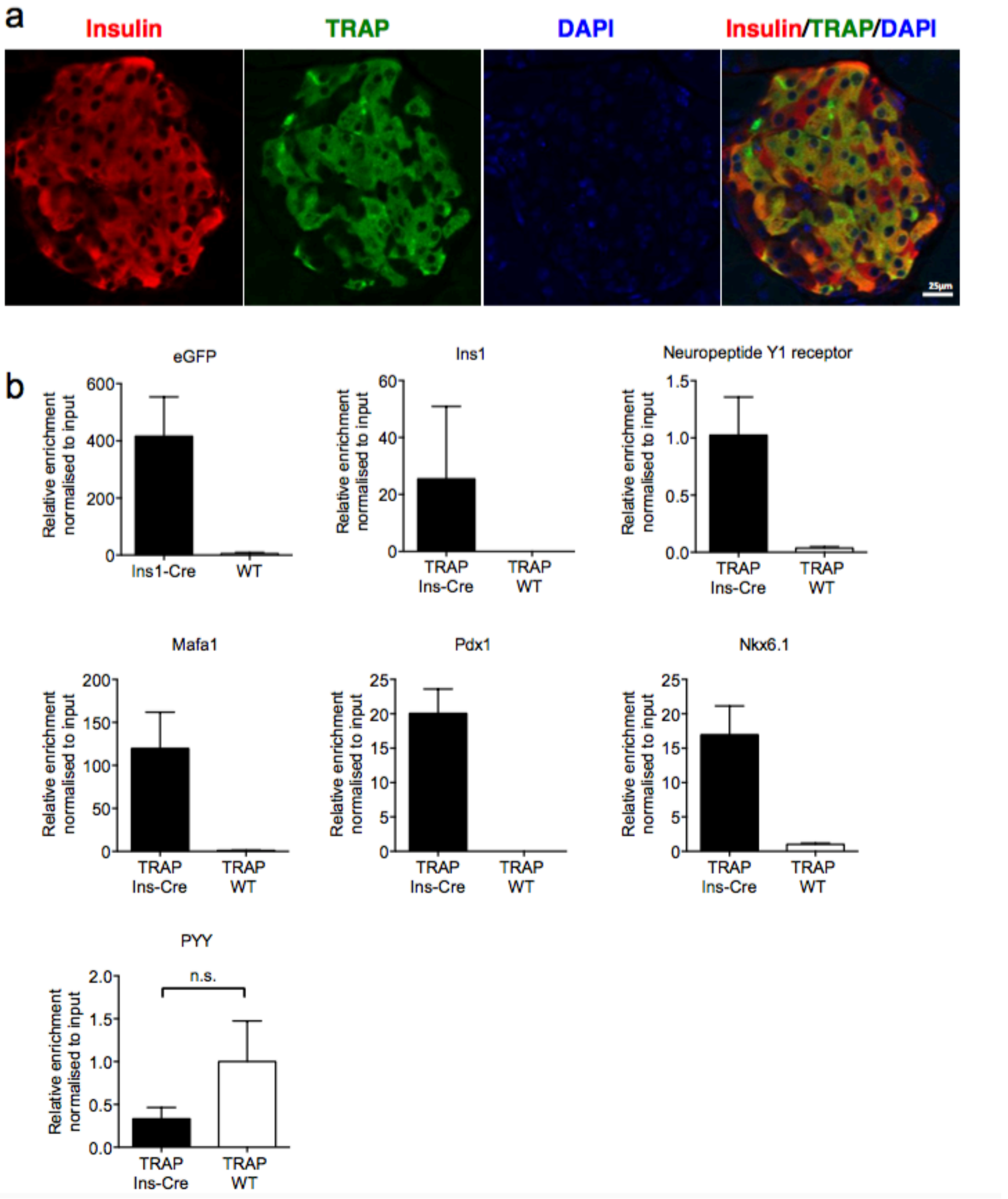

Supplementary Figure 1: Determination of Y1 receptor expression specifically in β-cells.

(**a**) Representative fluorescence micrographs showing an islet from a $Ins^{cre/+}$;TRAP mouse stained for insulin (red), TRAP (green) and nuclear counterstained with DAPI

(blue) and merged (n=4-5). (**b**) Enriched gene expression for *eGFP, Ins1, Mafa1, Pdx1, Nkx6.1 and Y1* receptor mRNA compared to TRAP negative islets. *PYY* was used as a negative control (n = 3-4 per group).

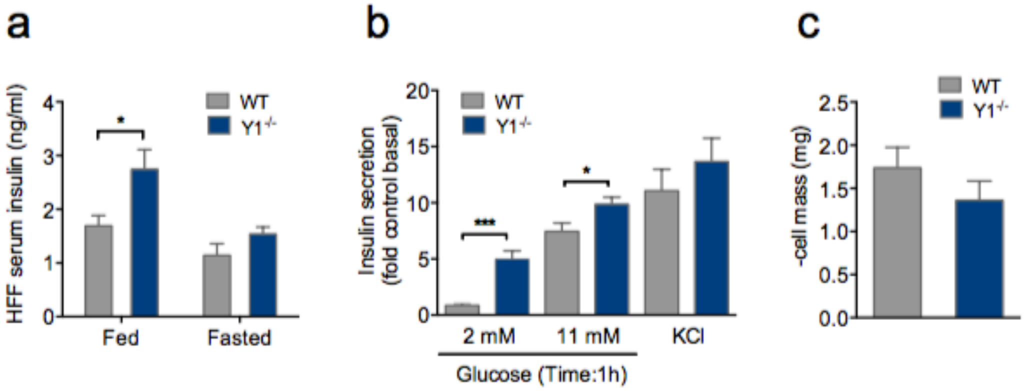

Supplementary Figure 2: Insulin response to β-cell specific Y1 receptor deletion.

(**a**) Fasting and fed serum insulin levels in β-cell specific Y1 receptor deletion mice (n = 8-10 per group). (**b**) Insulin release from isolated islets of β-cell specific Y1 receptor deletion in response to various concentration of glucose (n = 5). (**c**) Unaltered β-cell mass in 10 week-old $Y1^{-/-}$ mice. (n = 3). Data are means ± s.e.m. **P*<0.05, ***P<0.001 calculated by t-test analysis.

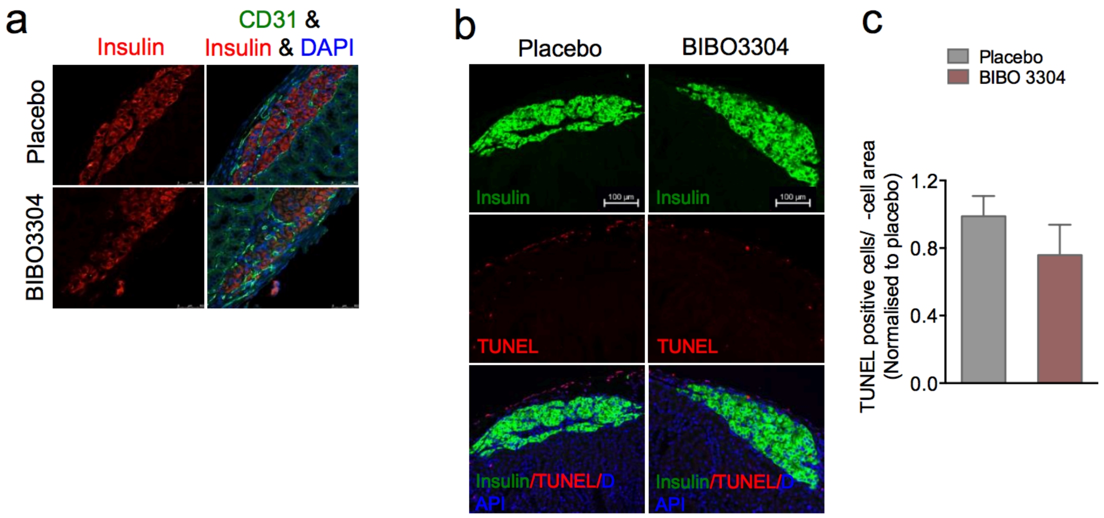

Supplementary Figure 3: Unaltered vascularization and apoptotic response in transplanted BIBO3304 treated islets.

(**a**) Representative fluorescence micrographs showing images of placebo and BIBO3304 treated islets stained for insulin (red), CD31 (green) and nuclear counterstained with DAPI (blue) (n = 4-5 per group). (**b**) Representative fluorescence micrographs showing images of placebo and BIBO3304 treated islets stained for TUNEL (red), insulin (green) and nuclear counterstained with DAPI (blue). (**c**) Quantification of TUNEL positive cell over β-cell area normalized to placebo group (n = 4). Data are means ± s.e.m. **P*<0.05, ***P*<0.01, ***P<0.001 calculated by t-test or two-way ANOVA analysis.

a

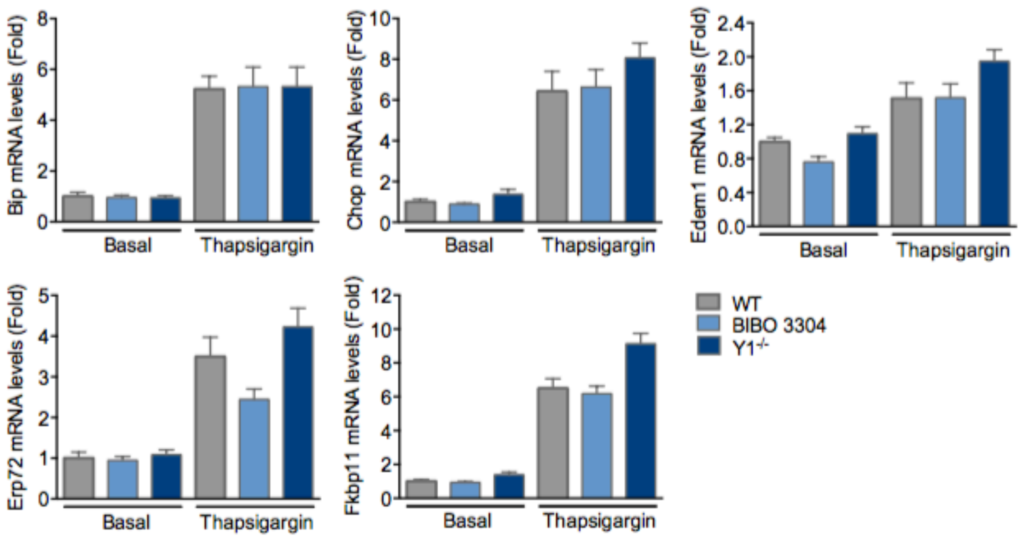

Supplementary Figure 4: Unaltered ER-stress responses in islets of WT, BIBO3304 treated and Y1$^{-/-}$ mice.

(**a**) Quantitative RT-PCR of ER stress markers in islets from placebo or BIBO3304 treated WT and Y1$^{-/-}$ mice at basal as well as in response to thapsigargin treatment (300 nM) for 24 h (n = 6 per group).

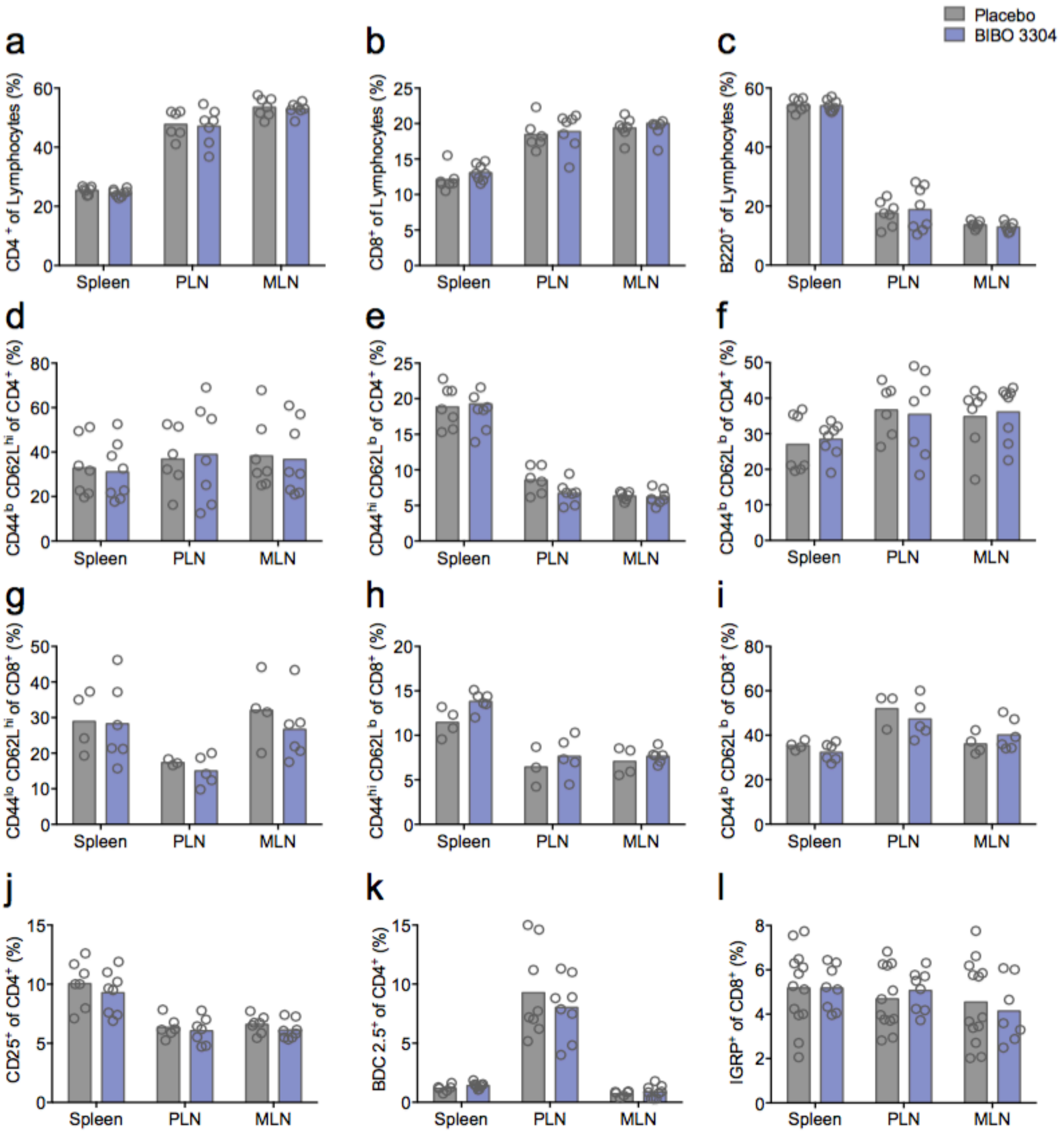

Supplementary Figure 5: Unaltered immune response in BIBO treated NOD mice.

Flow cytometric analysis of (**a**) CD4$^+$ T cells ($n > 6$). (b) CD8$^+$ T cells ($n > 6$). (c) B220$^+$ B cells ($n > 6$). (d) CD4$^+$ naïve T cells ($n > 6$). (e) CD4$^+$ memory T cells ($n > 6$). (f) CD4$^+$ effector T cells ($n > 6$). (g) CD8$^+$ naïve T cells ($n \geq 3$). (h) CD8$^+$ memory T cells ($n \geq 3$). (i) CD8$^+$ effector T cells ($n \geq 3$). (j) CD4$^+$ CD25$^+$ regulatory T cells ($n > 6$). (k) CD4$^+$ BDC 2.5$^+$ autoreactive T cells ($n > 6$). (l) CD8$^+$ IGRP$^+$ autoreactive T cells ($n > 6$).

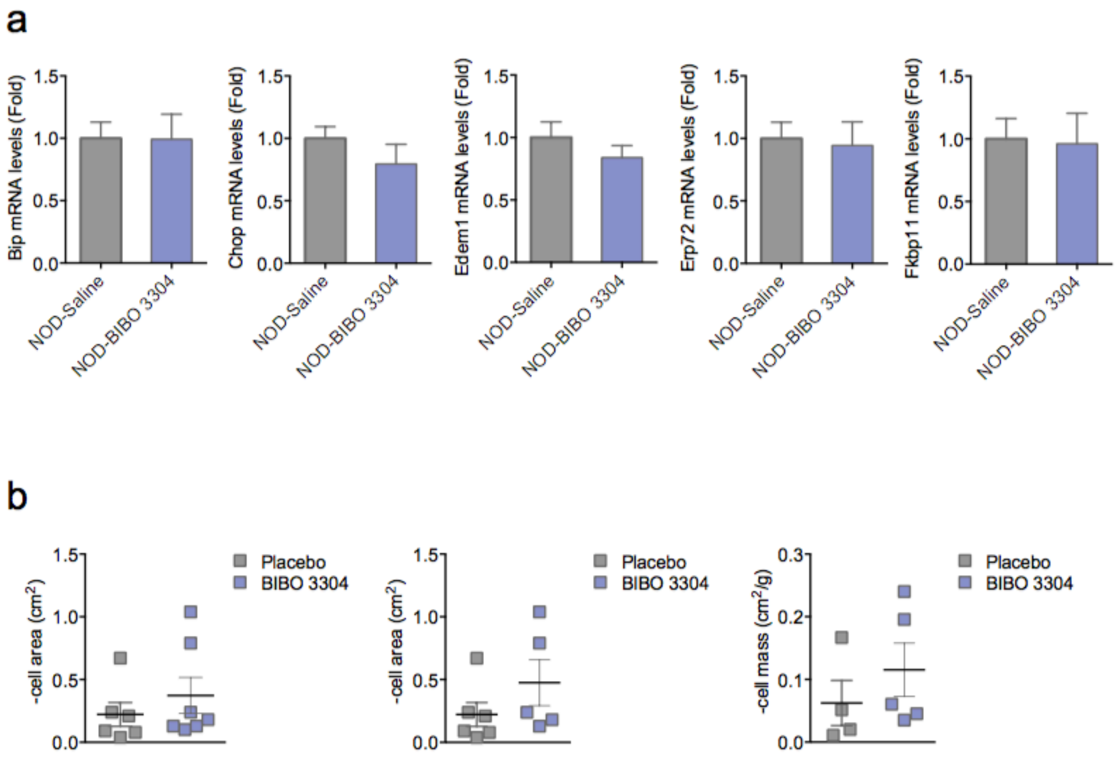

Supplementary Figure 6: ER stress markers and beta cell area of NOD mice treated with BIBO3304.

(**a**) Quantitative RT-PCR of ER-stress markers in islets from placebo or BIBO3304 treated NOD mice. (n = 6)

(b) Islet area of NOD mice treated with placebo (n = 6) and BIBO3304 (n = 7)

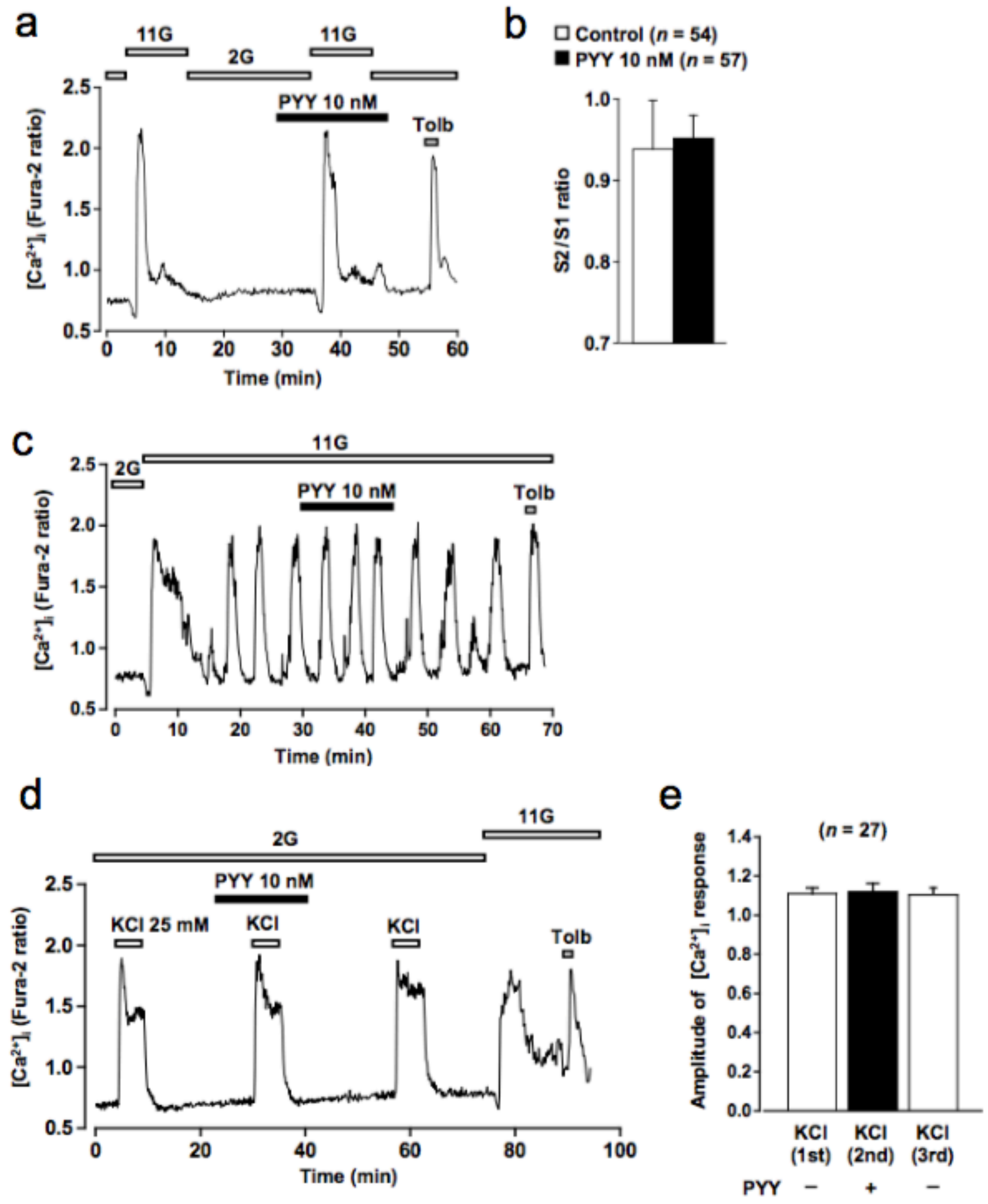

Supplementary Figure 7: Measurements of $[Ca^{2+}]_i$ in single β-cells. Cytosolic Ca^{2+} concentrations ($[Ca^{2+}]_i$) in mouse single β-cells were measured by dual-wavelength fura-2 microfluorometry.

(**a**) A repetitive stimulation with 11 mM glucose induced repeated $[Ca^{2+}]_i$ increases in β-cells. PYY (10 nM) did not affect $[Ca^{2+}]_i$ responses to 11 mM glucose. (**b**) The S2/S1 ratio of the peak amplitude of $[Ca^{2+}]_i$ response to the second glucose stimulation (S2) to that to the first stimulation (S1) was unaltered by PYY. PYY affected neither oscillations of $[Ca^{2+}]_i$ during the second phase responses to 11 mM glucose (**c**) nor KCl (25 mM)-induced $[Ca^{2+}]_i$ increases at 2 mM glucose in β-cells (**d,e**). Data were taken exclusively from the cells, which fulfilled the morphological and physiological criteria of β-cells including the diameter and responsiveness to glucose (11 mM) and K_{ATP} channel blocker tolbutamide (Tolb) (100 μM). Results are means ± s.e.m. (n=4-5).

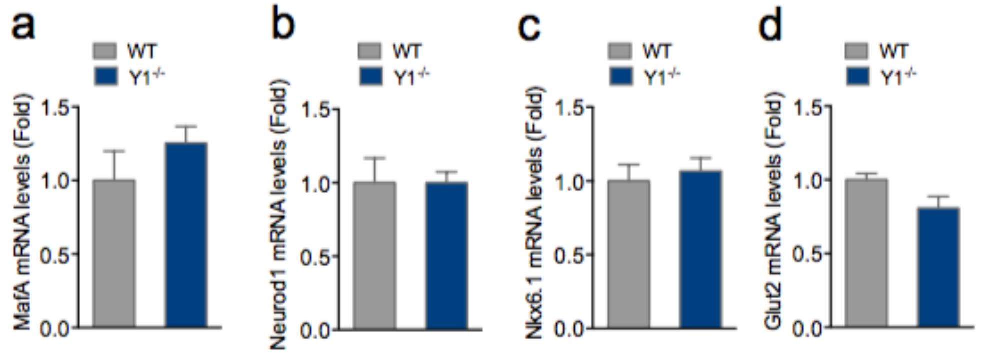

Supplementary Figure 8 Analysis of expression of genes that maintain β-cell phenotype. (**a-d**) Pancreatic islets from WT and Y1$^{-/-}$ mice were isolated and *MafA*, *Neurod1*, *Nkx6.1* and *Glut2* were determined using quantitative RT-PCR. *Rpl19* was used as a housekeeping gene. Results are means ± s.e.m. (n=3 mice per group).

Supplementary Table 1. Gene array analysis of Y-receptor expression in human islets

ProbeSetID	Average	Pcount	Symbol	Title
236860_at	3.18	0	*NPY6R*	Neuropeptide Y6 Receptor
207400_at	4.37	3	*NPY5R*	Neuropeptide Y5 Receptor
210729_at	3.01	0	*NPY2R*	Neuropeptide Y2 Receptor
205440_s_at	7.57	9	*NPY1R*	Neuropeptide Y1 Receptor
210956_at	5.25	0	*PPYR1*	Neuropeptide Y4 Receptor

Supplementary Table 2. Sequences of oligonucleotide primers (5'-3') used in qPCR

Gene symbol	5' Oligonucleotide	3' Oligonucleotide
Gk (Gck, MODY2)	CATTGAATCAGAGGAGGGCAGC	TAGTGGACTGGGAGCATTTGTGGG
Glut2 (Slc2a2)	CATTCTTTGGTGGGTGGC	CCTGAGTGTGTTTGGAGCG
Kir6.2 (Kcnj11)	TCGTGTCCAAGAAAGGCAACTG	GGAAGGCAGATGAAAAGGAGTGG
MafA	CGGGAACGGTGATTGCTTAG	GGAGGTTGGGACGCAGAA
mMdh (Mdh2)	AGGAAACCAGGAATGACACGGG	GAAAACTTCTGCTGTGATGGGG
Neurod1 (β2)	ACTCCAAGACCCAGAAACTGTC	ACTGGTAGGAGTAGGGATGCAC
Pc (Pcx)	GTTCCGTGTCCGAGGTGTAAAG	CGCAGAAGGATGTCCCTGAAAC
Sur1	CCTCAGCAGCACATTCCGTATC	CAGAAGCACAGCCAAGACATAGG
Tpi1	CCTTCCATTGGTTTGGGCTG	AATACAGGGGCTTTGGCACC
Vdccα1d	TAGCCACTCATTCCGCAACAC	GCAAGACCCTCAAAACCCTCAG
Cyclophillin (Cypa, Ppia)	TGTGCCAGGGTGGTGACTTTAC	TGGGAACCGTTTGTGTTTGG
Rpl-19	CTCGTTGCCGGAAAAACA	TCATCCAGGTCACCTTCTCA
Npyy1r	GGCCCACTCTGCTTTATAT	AGCAGCATGATGTTGATTCG
Nkx6.1	CCCGGAGTGATGCAGAGT	GAACGTGGGTCTGGTGTGTT
Pdx1	GAAATCCACCAAAGCTCACG	CGGGTTCCGCTGTGTAAG

PYY	CCTACCCTGCCAAACCAG	GGACATCTCTTTTTCCATACCG
Ins1	CTTCTTCTACACACCCATGT	ATCTACAATGCCACGCTTCT
eGFP	GAGCGCACCATCTTCTTCA	CGGATCTTGAAGTTCACCTT

Aliases of gene symbols given in parentheses
