# Supplementary material for: Inhibition of Y1 receptor signaling improves islet transplant outcome
Source: Nat Commun. 2017 Sep 8;8:490. doi: 10.1038/s41467-017-00624-2 (PMC5591241; doi:10.1038/s41467-017-00624-2)
Supplement: Supplementary file 2 — Peer Review file [file 41467_2017_624_MOESM2_ESM.pdf]

Editorial Note: *This manuscript has been previously reviewed at another journal that is not operating a transparent peer review scheme. This document only contains reviewer comments and rebuttal letters for versions considered at Nature Communications. Mentions of prior referee reports have been redacted.*

REVIEWERS' COMMENTS:

Reviewer #1 (Remarks to the Author):

This manuscript describes in considerable detail the role of PYY signalling via the Y1 receptor in beta cell function, quite clearly demonstrating a tonic inhibitory role of PYY on insulin secretion and a potential therapeutic benefit of Y1 receptor antagonism. The authors present compelling evidence for a beneficial application of Y1 receptor antagonism in islet transplantation. The data are extensive, clear, and support the conclusions.
